# An improved human anxiety process biomarker: characterization of frequency band, personality and pharmacology

**DOI:** 10.1038/tp.2015.188

**Published:** 2015-12-15

**Authors:** S M Shadli, P Glue, J McIntosh, N McNaughton

**Affiliations:** 1 Department of Psychology, University of Otago, Dunedin, New Zealand; 2 Department of Psychological Medicine, University of Otago, Dunedin, New Zealand

## Abstract

Anxiety disorders are among the most common mental illness in the western world with a major impact on disability. But their diagnosis has lacked objective biomarkers. We previously demonstrated a human anxiety process biomarker, goal-conflict-specific electroencephalography (EEG) rhythmicity (GCSR) in the stop-signal task (SST). Here we have developed and characterized an improved test appropriate for clinical group testing. We modified the SST to produce balanced numbers of trials in clearly separated stop-signal delay groups. As previously, right frontal (F8) GCSR was extracted as the difference in EEG log Fourier power between matching stop and go trials (that is, stop-signal-specific power) of a quadratic contrast of the three delay values (that is, power when stopping and going are in balanced conflict compared with the average of when stopping or going is greater). Separate experiments assessed drug sensitivity (*n*=34) and personality relations (*n*=59). GCSR in this new SST was reduced by three chemically distinct anxiolytic drugs (administered double-blind): buspirone (10 mg), triazolam (0.25 mg) and pregabalin (75 mg); had a frequency range (4–12 Hz) consistent with rodent model data; and positively correlated significantly with neuroticism and nonsignificantly with trait anxiety scores. GCSR, measured in our new form of the SST, should be suitable as a biomarker for one specific anxiety process in the testing of clinical groups and novel drugs and in the development of measures suitable for individual diagnosis.

## Introduction

Anxiety disorders are among the most common mental illness in the western world^1^ but their diagnosis^2, 3^ is not yet based on objective biomarkers. The new Diagnostic and Statistical Manual of Mental Disorders-5 has been criticized for its symptom-based diagnosis, with the US National Institute of Mental Health (NIMH) launching the Research Domain Criteria Project as an alternative syndrome-based system. The Research Domain Criteria Project lacks the key biomarker data.^4^ So, developing mental disorder biomarkers is urgently needed for accurate diagnosis and to meet global demand for effective treatment.

We have previously reported a human electroencephalography (EEG) biomarker, derived from the neuropsychological theory of the ‘Behavioural Inhibition System' (BIS),^5, 6, 7^ that identifies one specific anxiety-related process and so potentially one biological type of anxiety-related disorder.^8^ Here we report an improved means of eliciting the biomarker; clarify its frequency band; report its relations to measures of neuroticism and trait anxiety; and validate it with anxiolytic drugs of three different classes.

**Figure 1:**
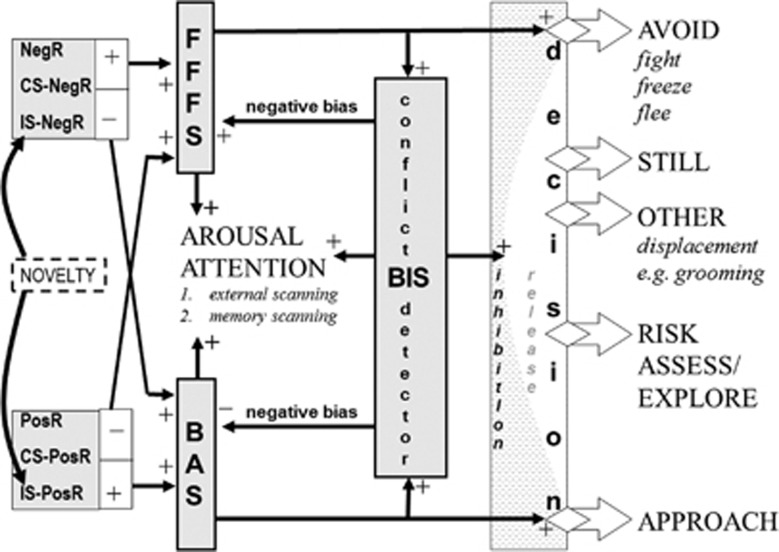
Overall relation of approach (BAS), avoidance (FFFS=fight, freeze, flee) and conflict (BIS=behavioural inhibition) systems—an updated model. The inputs to the system are classified in terms of the delivery (+) or omission (−) of primary positive reinforcers (PosR) or primary negative reinforcers (NegR) or conditional stimuli (CS) or innate stimuli (IS) that predict such primary events. The BIS is activated when it detects approach-avoidance conflict—suppressing prepotent responses and eliciting risk assessment and displacement behaviours. The systems interact in a variety of ways to generate behaviour, see text. The shaded areas are all points at which traits appear to operate. Figure and legend from McNaughton and Corr^13^ modified from Gray and McNaughton^6^ and Corr and McNaughton.^47^ BAS, behavioural approach system.

The BIS is defined as a system through which^9^ anxiolytics affect approach-avoidance conflict (anxiety-related) but not simple active avoidance (fear-related). It has a detailed, largely rodent-based, neuropsychology^5, 6, 7^ that assigns specific neural modules to specific aspects of fear and anxiety.^7^ However, for our present purposes, the BIS can be treated as a whole and as acting as a detector of conflict between approach goals and avoidance goals (Figure 1). With much higher levels of BAS (behavioural approach system) activation relative to FFFS (fight, flight, freeze system) activation, approach will occur or, vice versa, avoidance. However, the more equal and the stronger their activation, the more the BIS is activated—increasing arousal, attention and negative bias, while suppressing prepotent responding and replacing it with, for example, risk assessment. Importantly, anxiolytics reduce the output of the BIS, as a whole, via rhythmical slow activity (RSA; 4–12 Hz hippocampal rhythmicity). A reduction in electrically elicited RSA in rats predicts human clinical anxiolytic action with, so far, no false positives (even with sedatives) or negatives (even with drugs ineffective in panic or depression).^10^ Notably, RSA mediates anxiolytic action on behavioural inhibition in approach-avoidance conflict;^11^ and changes in rhythmicity, in and of itself, affect anxiolytic-sensitive behaviour.^12^ This specific interaction between anxiolytics and RSA in rats suggests that its human homologue could act as a clinical biomarker for an anxiety syndrome.

A human homologue of RSA was developed by Neo and McNaughton,^14^ Neo,^15^ and Neo *et al.*,^16^ and validated with anxiolytic drugs by McNaughton *et al.*^8^ They measured human scalp EEG during three task phases dominated by approach, conflict and avoidance, respectively. They subtracted the average power in approach and avoidance from that in conflict to measure goal-conflict-specific EEG rhythmicity (GCSR). Initial experiments using gain and loss of money to generate approach and avoidance with participants recruited from a student job-seeking pool found GCSR most consistently at F8.^14, 15^ Monetary reinforcement is likely to be problematic in the clinic and a range of forms of behavioural inhibition, including simple stopping, appear to share an orbital frontal locus of control.^17^ Neo *et al.*,^16^ therefore, used a stop-signal task (SST) that was identical to Aron *et al.*,^18^ who demonstrated control of stopping (an important output of the BIS) by the right inferior frontal gyrus. The key sole innovation made by Neo *et al.* to obtain GCSR was to sort trials during analysis into groups with short, medium and long stop-signal delays that resulted in more stopping, a balanced conflict between stopping and going, or more going, respectively. The procedure produced right frontal (F8) GCSR, consistent with a source in the right inferior frontal gyrus, that correlated with neuroticism and avoidance^16^ and was sensitive to both the anxiolytic benzodiazepine (GABA_A_ agonist), triazolam, and the anxiolytic 5HT_1A_ agonist, buspirone.^8^ So, right frontal GCSR elicited in the SST task in humans is pharmacologically homologous to RSA elicited by electrical stimulation in rats.

The SST involves no explicit threats and stopping behaviour does not correlate with neuroticism or trait anxiety^16^ and is not affected by benzodiazepine or 5HT1A drugs.^8^ Further, the speed of stopping (as measured by the stop-signal reaction time) allows little time for goal systems to exert rhythmic control and is completed half way through the period over which GCSR is assessed. GCSR in the SST, therefore, must involve slow goal-processing circuits, which operate in parallel^16^ with act and action circuits.^19^ This slower goal processing is sensitive to anxiolytic drugs, whereas faster motor control is not. So, although there is no explicit control of motivation in the SST, the accuracy and speed with which participants responded and stopped, coupled with the sensitivity of GCSR to the drugs that define the BIS, give us reason to believe that BAS and FFFS, and hence the BIS, are being activated in the SST to a similar extent to tasks using explicit monetary reinforcement.^14, 15^

However, in these previous experiments with the SST,^8, 16^ the frequency band for GCSR was variable and very narrow compared with rodent RSA, the three different groups of delays had no clear gap between them, and the sorting procedure generated unequal numbers of trials for the three different delays. In the current experiments, we modified the SST so as to directly control both the separation between short, intermediate and long delays and the number of trials within each delay group. We predicted this would produce statistically clearer changes in GCSR at F8. We found that this modified version of the SST spread GCSR through the range from 4 to 12 Hz in humans.

In Experiment 1, we tested the anxiolytic sensitivity of this broader band GCSR. Anxiolytic drugs, taken as a class that spans benzodiazepine, 5HT_1A_ agonists and calcium channel inhibitors, can ameliorate anxiety without also improving panic, phobia, depression or obsession.^20^ We tested members of each of these three distinct classes of anxiolytic: buspirone (5HT_1A_ agonist) and triazolam (GABA_A_ agonist) tested the replicability of their effects on GCSR; and Pregabalin (an α2-δ ligand that binds to an auxiliary protein associated with voltage-gated calcium channels) was tested on GCSR for the first time to increase generality. Experiment 2 confirmed that this broad band GCSR retained its relationship to personality.

Theta (4–7 Hz) and alpha (8–12 Hz) are usually considered functionally distinct in humans,^21^ but depth recording has shown that task-related human hippocampal slow waves could extend from the 4–7 Hz band to the 8–12 Hz band.^22, 23, 24^ Our finding of a broad range for human GCSR overlapping the conventional theta and alpha bands, and sensitive to three different classes of anxiolytic drug, is consistent with it being homologous with the rodent RSA (4–12 Hz) from which GCSR was theoretically derived.

## Materials and methods

### Participants

There were 59 participants (43 female,16 male; age 18–25 years) for the assessment of personality; and 40 participants (20 female, 20 male; with six excluded because of a computer failure) for the drug experiment. There were four different treatment groups in the drug experiment: placebo (three male, five female); buspirone (10 mg; five male, four female); triazolam (0.25 mg; four male, five female); and pregabalin (75 mg; four male, four female). The groups were balanced on entry (1:1:1:1) with a computer-generated block size of four. Buspirone and triazolam doses were the same as used previously^10^ with number of participants in sample chosen on the basis of this previous experiment. Pregabalin dose was based on the smallest available unit dose strength. Treatments were over-encapsulated to make them visually the same and were administered double-blind. After exclusions based on EEG artefact (see below) and computer failure, the groups remained approximately balanced. Participants were recruited from Student Job Search, reported no psychological disorders and they were not taking any drug related to mental disorders. They provided written consent before the experiments; with consent for the drug experiment undertaken by a psychiatrist (PG). They each received NZ$30 as a reimbursement in recognition of the inconvenience and costs related to taking part in the study. The experiments were approved by the University of Otago Human Ethics Committee (approval numbers: DP 10/07 and 13/035).

### Procedure

Participants filled out the EPQ-R (Eysenck Personality Questionnaire - Revised),^25^ BIS/BAS scale^26^ and STAI (Spielberger State-Trait Anxiety Inventory)-Trait questionnaires^27^ after arrival at the laboratory. For the drug experiment, they were first administered white-coated capsules before filling out the questionnaire. The participants were then prepared for EEG recording. After arrival on the test date, the experimenter measured their head circumference and marked Fp1 and Fp2 according to the International 10–20 system with a black marker. The appropriate-sized cap depending on the participants head circumference was then selected for each participant and fitted to their head. A syringe and blunt square-tipped 16-gauge needle (Precision Glide Needle, Becton Dickinson, Franklin Lakes, NJ, USA) was used to inject conducting gel (Electro-Cap International, Eaton, OH, USA) and the impedance of each electrode was reduced by careful abrasion with the tip of the needle to achieve 5⩾KΩ (completion time ranged between 30 and 50 min) for behavioural and drug study. Participants were then seated in the chair and their cap connected to the EEG machine. We used two different EEG recording systems for the two different experiments. For the personality experiment, we used an Electro-Cap International with an eight-channel BioRadio (Cleveland Medical Devices, Cleveland, OH, USA) recording system. For the drug experiment, we used a Waveguard (Ag/Agcl) cap with a 32-channel ASA Neurotechnology system (ANT Neuro, Enschede, The Netherlands). The sampling rate was 128 Hz, band pass was 1–36 Hz, and impedance was reduced to below 5 KΩ. Once acceptable impedances were obtained, deliberate eye-blink traces and relaxation-induced alpha rhythm were assessed to screen for oddities in the recordings and further electrode adjustments made where necessary. The STAI-State questionnaire was then administered, followed by the SST task. Immediately after the SST task, they were given the STAI-State questionnaire again. For the drug experiment, participants started the SST 60 min after taking their capsules, when peak blood levels would be anticipated.

### Stop-signal task

The SST used in the current experiments was a further development of the SST in our previous experiments^8, 16^ based on Aron *et al.*^18^ It had the following modifications: (1) short and long, but not intermediate, delays were controlled by Go response time using the method of Carter *et al.*;^28^ (2) colour was added to increase the discriminability of the Go stimuli; (3) smiley/frowney face feedback on correct/incorrect performance was added after each trial to increase motivation for correct responding; (4) ‘slow' feedback was provided when the participant's Go reaction time during Stop testing exceeded 1.5 times their reaction time in pretesting with pure Go trials to reduce strategic slowing.

Separate control of short and long SSDs solved two structural problems with the original SST. First, there was an imbalance between short, medium and long trial numbers as these were simply sorted into three groups with no control over group size. Second, there was no clear division between the three groups, as their SSD values were contiguous. Both unequal numbers and a lack of clear division were statistically undesirable if differences between the groups of trials were being tested. The more extreme separation of the short and long SSDs, in the event, solved a third problem. The SST based on Aron *et al.,*^18^ used by Neo *et al.*,^16^ found GCSR only at 7–8 Hz and a slightly modified version^8^ found GCSR only at 9–10 Hz. The rodent model on which GCSR is based generally finds RSA to have a much broader frequency spectrum ranging from 4 to 12 Hz frequency.^6, 29, 30^ As shown in results, widening the gap between short and long SSDs (sampling across a greater extent of the underlying inverted-U conflict function) produced wider band GCSR.

In the standard SST, there is no indication as to whether responding is on the correct button or whether stopping has been successful. The addition of ‘smiley and frowney' feedback was intended to increase the motivational value of correct and incorrect responding and so, also, the intensity of conflict. Similarly, the time limit ‘slow' feedback was intended to reduce the tendency of some people to steadily slow their Go response to try and succeed in stopping. This was intended to increase the stability of responding and reduce variation in Go reaction times.

**Figure 2:**
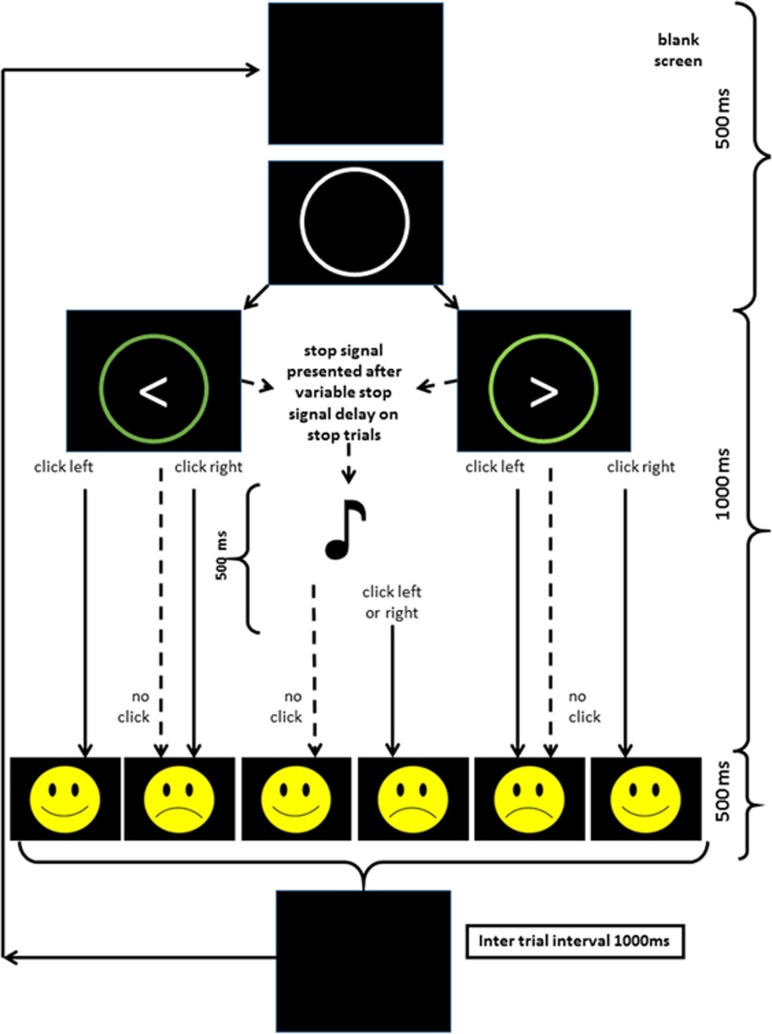
Events in the stop-signal task. Each trial starts with a blank screen that turns into a white fixation circle. The fixation circle then turns green when the go signal (either left or right arrow) is presented. This is occasionally followed by a stop signal (auditory tone). Depending on the participant's response, they were then presented with feedback of either a smiley or a frowney face as indicated.

On Go trials, a white fixation circle was presented on the centre of the screen against a black background, followed by a left/right white arrow that appeared in the circle 500 ms later. Participants were instructed to press the left/right mouse click as quickly and accurately as possible in response to the left/right arrow, respectively. On Stop trials, the stop signal (a tone) was presented at variable delays and participants were told to withhold their mouse click on these trials. The SST task consisted of three blocks each of 128 trials, with a stop signal being presented once in every four trials, so each block contained 32 Stop trials and 96 Go trials. Further details, stimulus images, and a schematic of the procedure, are shown in Figure 2.

Within each 128-trial block, the stop-signal delays (SSDs) were systematically varied between trials. This was controlled using a staircase-like tracking system. This modified SST had three nominal ‘staircases' delivering short, medium and long SSDs. The short and long SSD values were set to 20% and 80%, respectively, of the average GO reaction time over the previous 16 Go trials. The medium staircase was set to start at 45% of pre-training Go reaction time but then tracked responding (increasing after successful stopping and decreasing after failed stopping) as with the staircases used by Aron and Poldrack^31^ and Neo *et al.*^16^ but in 30 ms rather than 50 ms steps and with a restriction that the SSD could never get closer than 50 ms to the current value of either of the other staircases. The intermediate staircase was expected, therefore, to track the 50% correct stopping point where maximum conflict is expected in the BIS theory.

### Data analysis

Residual mains noise was filtered using a simple three-point running mean with an effective cut-off of 43 Hz. Eye blink artefacts were removed, leaving residual EEG, by automatically fitting a template of the ballistic components of each eye blink to activity at Fp1 and then subtracting this from other channels after scaling with a least squares technique.^16, 32^ Then, the experimenter removed artefacts that were not detected by the automated procedure, replacing them with missing values.

A 1-s Hanning window was used with 0.25 s before the stop signal on Stop trials. The Hanning window is a cosine wave applied to the 1 s and so extracts maximum power from the middle 0.5 s period, and least power from the leading and trailing 0.25 s periods. This doubles the frequency resolution of the subsequent Fourier transform compared with a 0.5 s square window. The procedure was essentially the same for matching Go trials, except that the window was located in relation to where the stop signal was presented in the immediately adjacent stop trial. The data were then Fast Fourier Transformed, log transformed to normalize error variance, and averaged across trials for that participant. Our data analysis was limited only to channels F7, F3, Fz, F4 and F8, with the focussed analysis presented in the main body of the paper (based on our previous results) being restricted to F8.

### Statistical analysis—analysis of variance

Factors of interest that were included in the analyses were the SSD (early, intermediate, late), frequency (4–12 Hz, in the 1-Hz increments resulting from the Fourier transform), trial type (Stop, Go) and the three trial blocks. Primary analysis was restricted to the right frontal channel, F8 as Neo *et al.*^16^ observed neuroticism and trait anxiety correlations with GCSR power only at F8, and McNaughton *et al.*^8^ found the clearest anxiolytic drug effects at F8. So, to maintain maximal statistical power, our primary analysis was limited to the F8 electrode, with subsequent comparisons across all frontal channels to assess specificity to F8.

Analyses of variance were performed using the IBM SPSS package 21 (IBM North America, New York, NY, USA). The central statistic of interest based on our hypotheses was the EEG power when two goals were equally activated (that is, stopping versus going). Effects specific to stopping were assessed as the difference in power between Stop and Go trials (Stop–Go) in the 0.5 s duration of the stop signal. The effects of SSD were assessed via orthogonal linear and quadratic contrasts^33^ with the short, intermediate and long SSD trials as successive levels. Mathematically, in this three-level case, the linear contrast of the different SSD conditions tested the amount of variation between the short and long conditions independently of the intermediate one, and the quadratic contrast was the difference between the intermediate and the average of the short and long trials—that is, conflict-specific changes. Any frequency-related changes across 4–12 Hz were also assessed with linear and quadratic contrasts with 4, 5, 6, 7, 8, 9, 10, 11 and 12 Hz as successive values. All the *P-*values reported are uncorrected unless stated otherwise.

## Results

### Experiment 1—behavioural measures

Demographic and behavioural data for the different drug groups of experiment 1 are shown in Table 1. There were no significant variations between the groups observed, indicating no sampling biases. Neuroticism, BIS and trait anxiety scores were almost identical between the groups. As previously,^8^ the drugs did not have any noticeable effects on any SST behavioural measures.

### Experiment 1—drug effects on GCSR at F8

**Figure 3:**
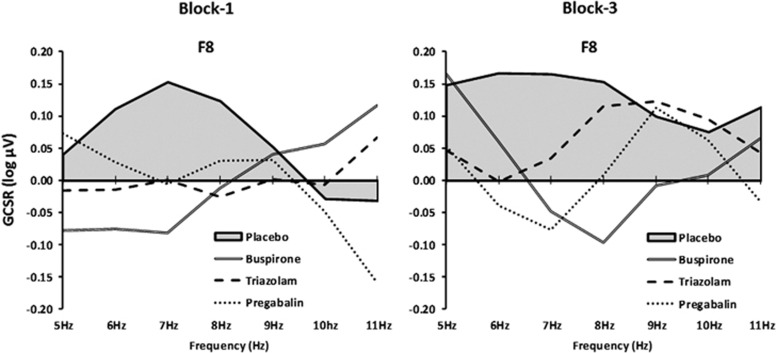
Frequency variation in the conflict effect at F8, assessed as the difference between Stop–Go power for the medium SSD trials and the average of the contrasts for the short and long SSD trials for the four different drug groups. The positive grey shaded area represents the predicted conflict effect in the placebo group—all the three treatments reduced this effect at 5–9 Hz in both the blocks. GCSR, goal-conflict-specific rhythmicity; SSD, stop-signal delay.

We observed GCSR at F8 in the placebo group in both block 1 and block 3 (Figure 3) but not in block 2 and not at F7, F3, Fz or F4 in any block (data not shown). This pattern was repeated in experiment 2. All the three drugs reduced GCSR in block 1 (Stop–Go × SSD × frequency × group, linear × quadratic × linear × group, F(3,36)=3.525, *P*<0.05) and block 3 (Stop–Go × SSD × frequency × group, linear × quadratic × linear × group, F(3,36)=5.383, *P*<0.01). They did not produce equivalent power reductions in block 2 (data not shown).

Comparison with the other channels showed that there were maximal GCSR and drug effects at F8 with opposite or much lesser effects progressing through F4, Fz, F3 to F7 in both block 1 (Stop–Go × SSD × frequency × channel × group, linear × quadratic × cubic × linear, F(3,36)=2.84, *P*<0.05) and in block 3 (Stop–Go × SSD × frequency × channel × group, linear × quadratic × linear × linear, F(3,36)=3.153, *P*<0. 05).

### Experiment 2—frequency range and personality correlations

Demographic and behavioural data for the participants of experiment 2 are shown in Table 2. The main result of experiment 1 was that, consistent with our previous experiments, a positive GCSR was observed at F8. However, the increases in power were larger than previously and spanned a wider frequency range: 4–12 Hz. This was observed during the first block of 128 trials (block 1), disappeared during the second block of 128 trials (data not shown) and then reappeared to some extent in block 3.

**Figure 4:**
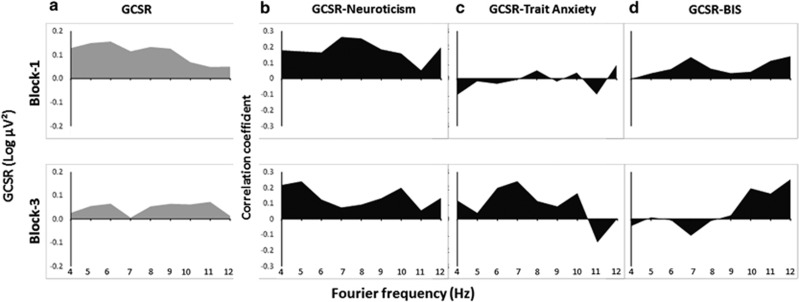
(**a**) Variation in GCSR power with frequency at F8, averaged across all the participants. The value plotted was obtained by first subtracting power at the matching point in the preceding Go trial from power in the Stop trial and then taking this value for medium SSD trials and subtracting the average Stop–Go value for the short and long SSD trials. (**b**) Correlation between GCSR at F8 and neuroticism scores (EPQ-R). (**c**) Correlation between GCSR at F8 and trait anxiety scores. (**d**) Correlation between GCSR at F8 and BIS scores. BIS, behavioural inhibition system; GCSR, goal-conflict-specific rhythmicity; SSD, stop-signal delay.

Figure 4a shows the variation in GCSR power (that is, the contrast of Stop–Go × quadratic of SSD) with frequency and training block. The predicted increases in power at F8 were seen in block 1 (Stop–Go × SSD × frequency, linear × quadratic × cubic, F(1,59)=5.38, *P*<0.05) and in block 3 (Stop–Go × SSD × frequency, linear × quadratic × linear, F(1,59)=4.01, *P*<0.05).

Figures 4b–d show the related variation in the correlations of F8 GCSR power with neuroticism, trait anxiety and BIS scores, respectively. For block 1, GCSR showed positive correlations with neuroticism scores at all the frequencies; little obvious systematic relation with trait anxiety scores; and showed consistently positive but individually modest correlations with behavioural inhibition scores. For block 3, neuroticism was less strongly but still consistently positively related to GCSR, the bulk of the trait anxiety correlations were positive; and behavioural inhibition correlations were more positive than negative. Stepwise multiple regression detected a significant relation of GCSR only with neuroticism at 7 Hz in block 1 (*r*=0.264, F_change_=4.254, df=1/57, *P*<0.05).

## Discussion

These experiments demonstrate GCSR ranging from 4 to 12 Hz at F8 that is sensitive to three distinct classes of anxiolytic drugs. In both frequency band and pharmacology, GCSR is homologous with the rat hippocampal RSA from which GCSR was theoretically derived. GCSR also correlated, as expected, with questionnaire measures of factors thought to predispose to (neuroticism) or directly reflect (trait anxiety) high levels of anxiety. GCSR, measured in our new form of the SST, should be suitable as a biomarker for one specific type of anxiety disorder in one-off testing of groups but not diagnosis of individuals or test–retest comparisons (see below).

Three different classes of anxiolytic drugs (which share only anxiety-specific action and do not share actions on panic, obsession, depression or side effects) all reduced GCSR with a single acute dose. Buspirone (a 5HT1_A_ agonist) and triazolam (GABA/benzodiazepine agonist) can relieve clinical anxiety^8, 34, 35, 36^ while not affecting panic or depression, respectively. Pregabalin (an α2-δ ligand associated with voltage-gated calcium channels), which we believe we show for the first time here reduces GCSR, can relieve clinical anxiety while not affecting either panic or depression.^37, 38, 39^ This common action of widely different classes of anxiolytic replicates our previous narrow-band results^8^ and validates this broader band GCSR as an anxiety process biomarker.

GCSR has a strong pre-clinical neuropsychology^29^ and, particularly with our improved SST, should be one of the first mental disorder biomarkers of the type required by the Research Domain Criteria Project approach proposed by NIMH.^4^ The precise nature of the process that gives rise to GCSR (and so the precise nature of the disorder for which it is a biomarker) requires further work. However, the process giving rise to GCSR is unlikely to contribute strongly or directly to panic, phobia, obsession or depression. Extreme GCSR, then, is likely to be a biomarker for a generic form of anxiety disorder.

All three anxiolytics reduced GCSR compared with placebo with a single acute administration, as predicted. This acute action matches rodent experiments with RSA,^10^ where long-term administration of anxiolytics does not change their effects.^40^ Unlike with human clinical anxiety, acute dosing is also effective in behavioural tests in rodents where the behaviour is hippocampal-dependent;^34^ or anxiety is an immediately elicited state;^41^ or anxiety is in the process of being learned.^9^ GCSR appears, therefore, to reflect a process that generates acute anxiety and supports the development of chronic anxiety so that its reduction with drugs results in a steady, extinction-like decline in clinical anxiety. Importantly, these results show that GCSR could be used in single-dose human screening of novel classes of anxiolytic drugs. It (like rodent RSA) shows an immediate response to all classes of anxiolytics (5HT1_A_, GABA_A_ agonist and calcium channel inhibitor) with only a single acute dose. This single acute dosing would hugely decrease the cost and increase the certainty for screening novel compounds in humans.

There are major limitations, however, to its use as an individual diagnostic instrument. First and foremost is that its occurrence appears transient. In previous experiments with humans reinforced with money, GCSR was observed only during initial learning and not when differential responding was fully established and had stabilized.^14^ This is as expected from rodent experiments where chronic anxiolytic treatment affects learning of behavioural inhibition but does not affect it when it is well established.^42^ In the current experiments GCSR also appeared transiently and, although we have not tested this directly, we would expect it not to show test–retest reliability or, indeed, occur substantially in a retest at all. In all these cases, we would see the BIS as being involved only when motivated goal control is predominant and to show minimal involvement once the action is habitual.

Broad-band GCSR correlated significantly with neuroticism and, perhaps less so, with trait anxiety, consistent with previous narrow-band GCSR results. This pattern is consistent with the suggestion that high neuroticism is a risk factor for anxiety.^43^ These results are also consistent with previous linkage of right frontal power to personality measures thought to link to the BIS.^14, 44, 45, 46^ However, we failed to find any strong relationship of GCSR with the ‘BIS' scale (which was derived psychometrically without any biological anchoring). The small size of our sample (*N*=59) allows only qualitative and not quantitative conclusions to be drawn about the personality measures. However, the data suggest that GCSR could be used in the development of questionnaire or other measures suitable for individual diagnosis.

GCSR, as measured with the current methods, would not be effective for individual diagnosis. It lacks both sufficient accuracy and test–retest stability. EEG is highly variable and so GCSR is a better measure for group than individual data analysis. Likewise, the current SST is a brief challenge test and so is unlikely to generate reliable repeat readings. However, GCSR has the capacity to characterize groups of people (it distinguishes clearly between placebo and low-dose anxiolytic treatment with *N* in group less than 10). It also correlates with reliable and stable personality measures. GCSR should, therefore, be able to act as an anchor for the development of a future individual diagnostic tool.^29^

In sum, we report here a human EEG rhythmicity biomarker that is homologous in terms of frequency band and pharmacology to the rodent test from which it was derived; is sensitive to three classes of anxiolytic (which as a group share only anxiolytic and not panicolytic, anti-obsessional or antidepressant actions or any side effects); correlates with anxiety-related measures of human personality; has the power to detect differences in groups owing to clinical condition or drug treatment; and could be used to develop individual diagnostic instruments.

## Figures and Tables

**Table 1:** Average demographic data (s.d. in brackets) for the different drug groups in the three-staircase auditory SST in experiment 1

*Group*	*Age*	*M/F*	*EPQ*		*BIS*	*STAI-T*	*GO_RT*	*SSRT*	P_*inhibit*_ *%*		
			*Neur*	*Extr*					*Short*	*Med*	*Long*
Placebo	21 (2)	3/5	13 (6)	3 (1)	18 (2)	39 (2)	426 (19)	238 (26)	79 (9)	51 (2)	6 (3)
Buspirone	20 (2)	5/4	12 (5)	6 (2)	19 (2)	41 (3)	410 (51)	230 (38)	70 (10)	49 (3)	5 (2)
Triazolam	22 (2)	4/5	13 (5)	5 (2)	16 (3)	36 (3)	428 (38)	237 (31)	76 (12)	52 (4)	8 (3)
Pregabalin	21 (1)	4/4	12 (6)	7 (3)	20 (3)	37 (2)	411 (44)	226 (22)	74 (13)	51 (4)	4 (2)

Abbreviations: BIS, scores on the behavioural inhibition questionnaire; Extr, EPQ extraversion; Go_RT, go reaction time on Go trials in ms; Med, intermediate; M/F, number of male and female participants per group; Neur, EPQ neuroticism; *P*_inhibit_, probability of inhibition on Stop trials; SSRT, stop-signal reaction time (mean stop-signal delay on the intermediate staircase subtracted from median Go reaction time); SST, stop-signal task; STAI-T, Spielberger trait anxiety. There were no statistical differences between the groups.

**Table 2:** Average demographic data (s.d. in brackets) for the three-staircase auditory SST in experiment 2

*Age*	*M/F*	*EPQ*		*BIS*	*STAI-T*	*GO_RT*	*SSRT*	P*_inhibit_ %*		
		*Neur*	*Extr*					*Short*	*Med*	*Long*
21 (4)	16/43	9 (6)	13 (4)	21 (8)	38 (8)	425 (42)	240 (25)	79 (11)	49 (6)	9 (4)

Abbreviations: BIS, scores on the behavioural inhibition questionnaire; Extr, EPQ extraversion; Go_RT, go reaction time on Go trials in ms; Med, intermediate; M/F, number of male and female participants per group; Neur, EPQ neuroticism; *P*_inhibit_, probability of inhibition on Stop trials; SSRT, stop-signal reaction time (mean stop-signal delay on the intermediate staircase subtracted from median Go reaction time); SST, stop-signal task; STAI-T, Spielberger trait anxiety. There were no statistical differences between the groups.
